# Supplementary material for: Litter Decomposition in Pacific Northwest Prairies Depends on Fire, with Differential Responses of Saprotrophic and Pyrophilous Fungi
Source: Microorganisms. 2025 Aug 6;13(8):1834. doi: 10.3390/microorganisms13081834 (PMC12388238; doi:10.3390/microorganisms13081834)
Supplement: Supplementary file 1 [file microorganisms-13-01834-s001.zip › microorganisms-3728861-supplementary.pdf]

Supplementary Materials

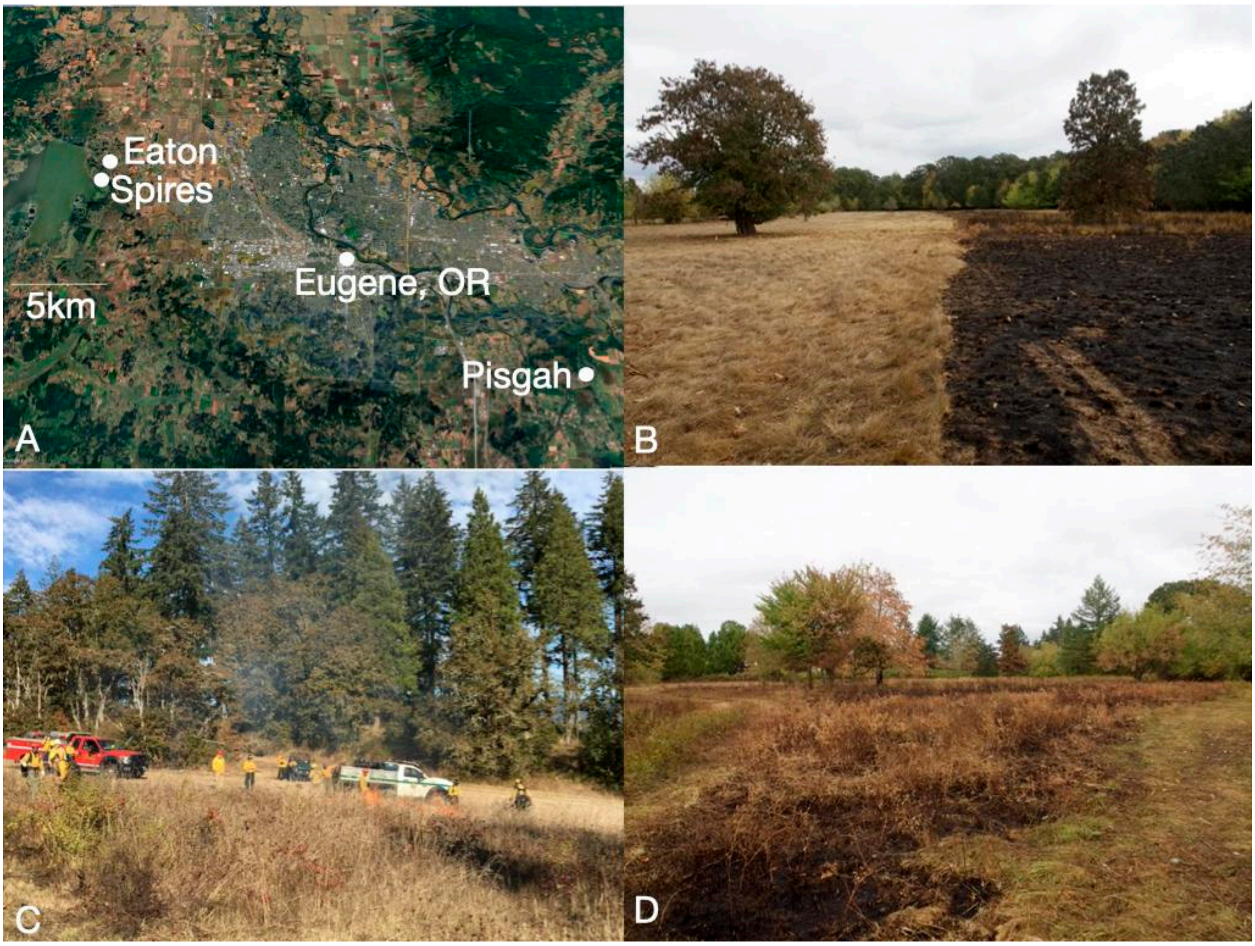

Figure S1. Sites. (**A**). The prairie sites are marked on a Google Earth image, which shows that all the sites are within 15 km of Eugene, Oregon. (**B**). Eaton, showing a 2016 burn. (**C**). Pisgah showing the October 2022 burn in progress. (**D**). Spire showing a 2016 burn. Photos (**B**) and (**D**) by Bitty Roy and photo (**C**) by Ed Alverson, used with permission.

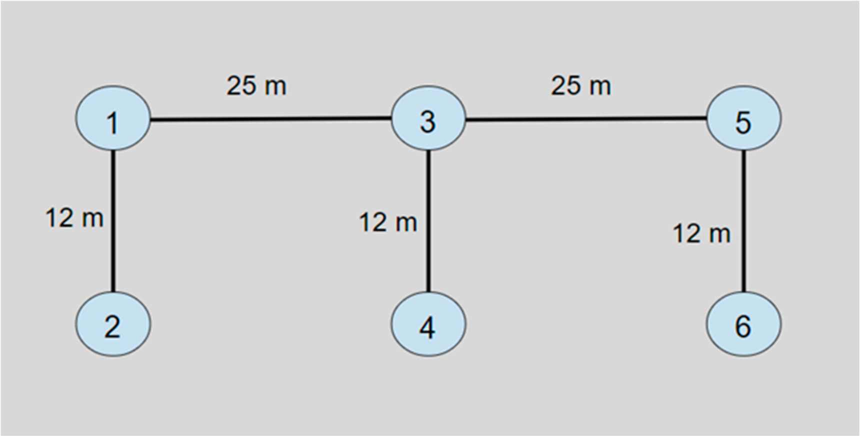

Figure S2. Litter bag plot layout. Each unburned vs. burned meadow was laid out the same. The litter bags were thus randomly placed with respect to the environment they were in and there was replication both within and among

sites. Four autoclaved and four untreated bags were left at each numbered point. Numbers 1-6 represent sample numbers.

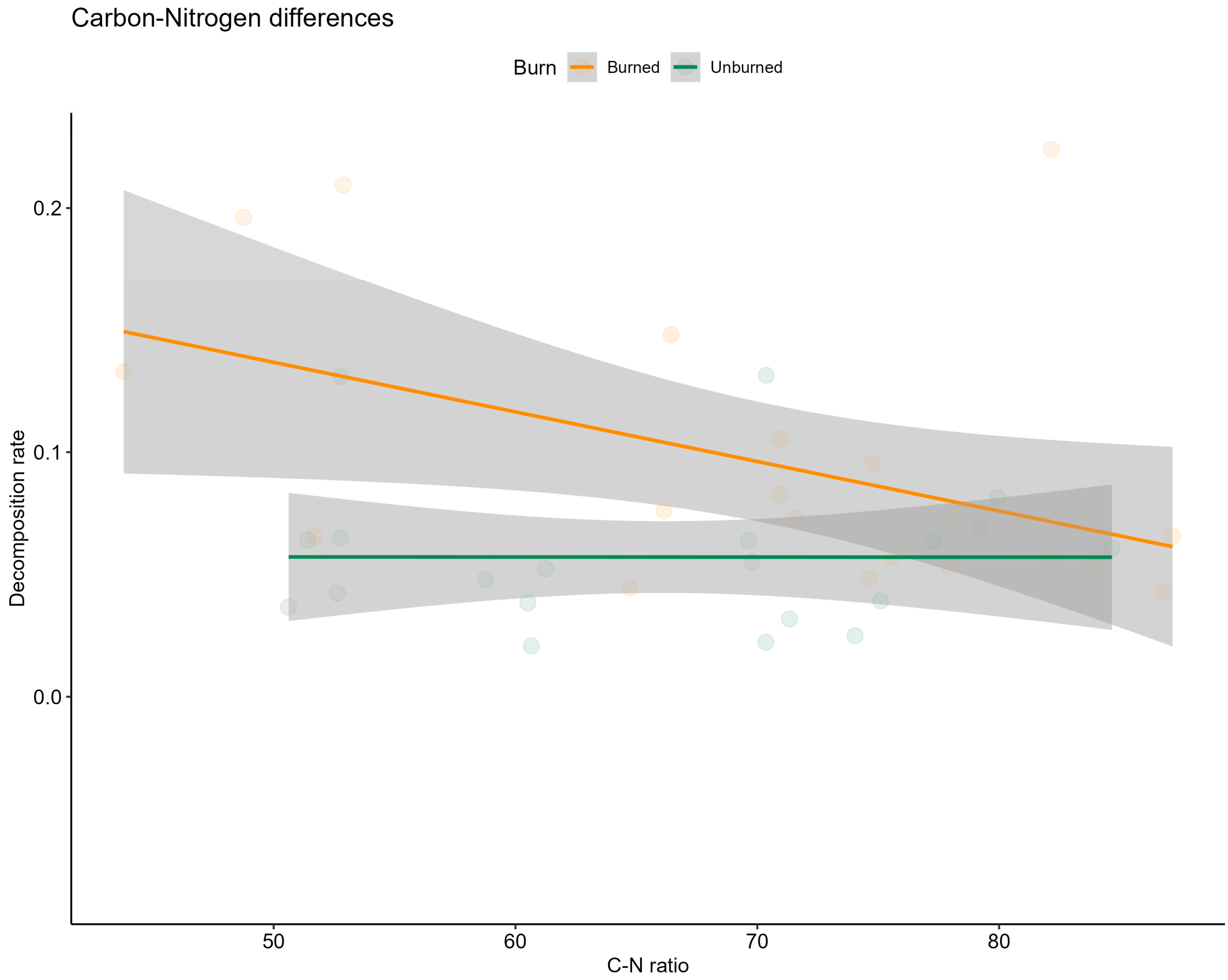

Figure S3. Carbon to Nitrogen ratio in burned vs. unburned litter. Decomposition rate decreased as C-N increased (*p* = 0.01), there was no significant effect of treatment.

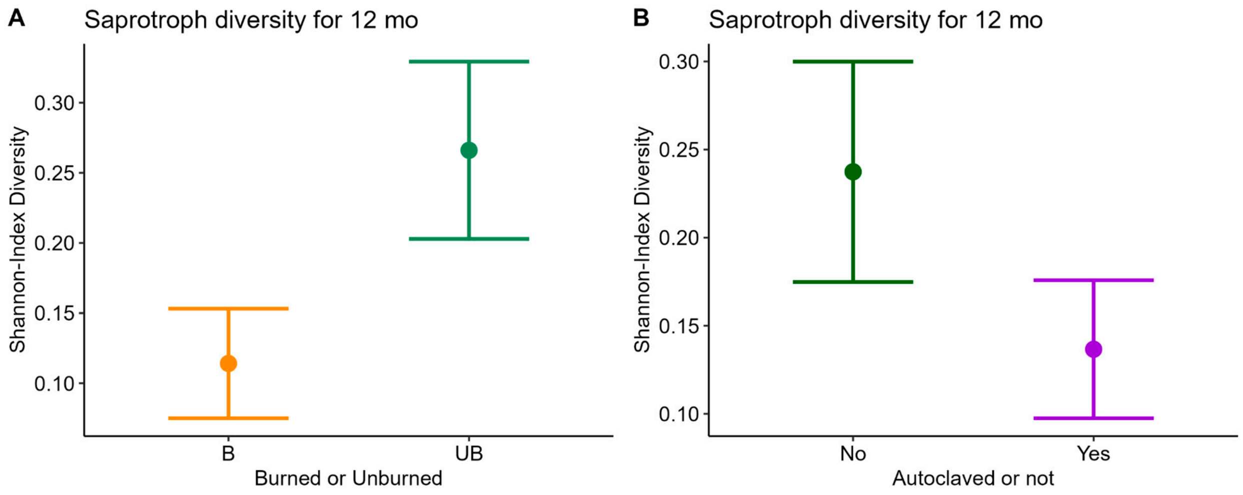

Figure S4. Saprotroph diversity at just 12-month sampling. These data include Pisgah sites, showing that by the end of the year, saprotroph diversity was

higher in unburned sites than burned (p = 0.06, (**A**)) and trending higher in untreated litter than autoclaved (p = 0.12, (**B**)).

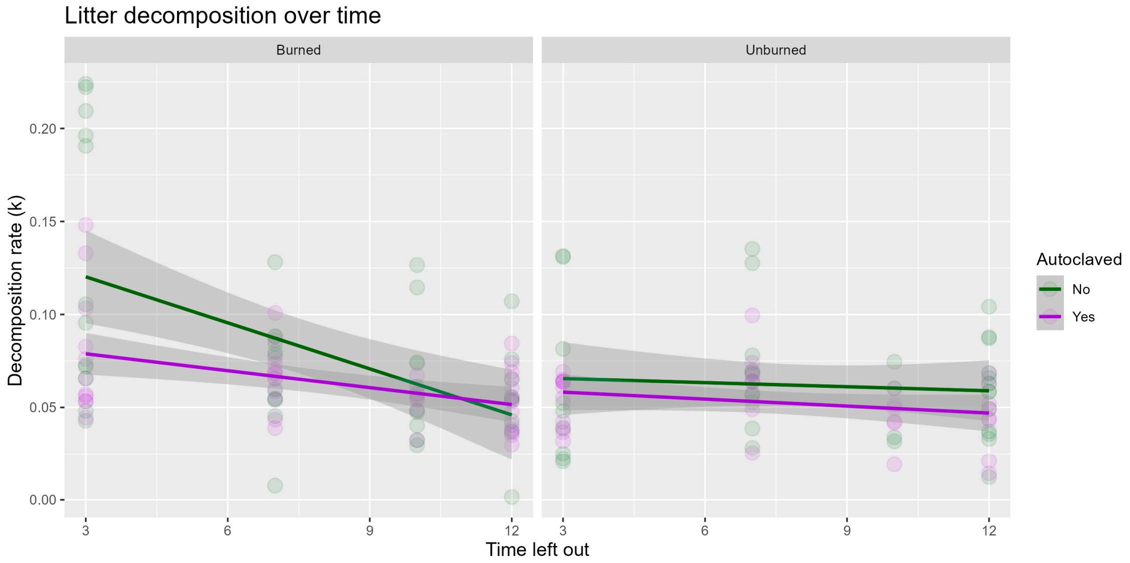

Figure S5. Litter decomposition over time, with Pisgah sites removed.

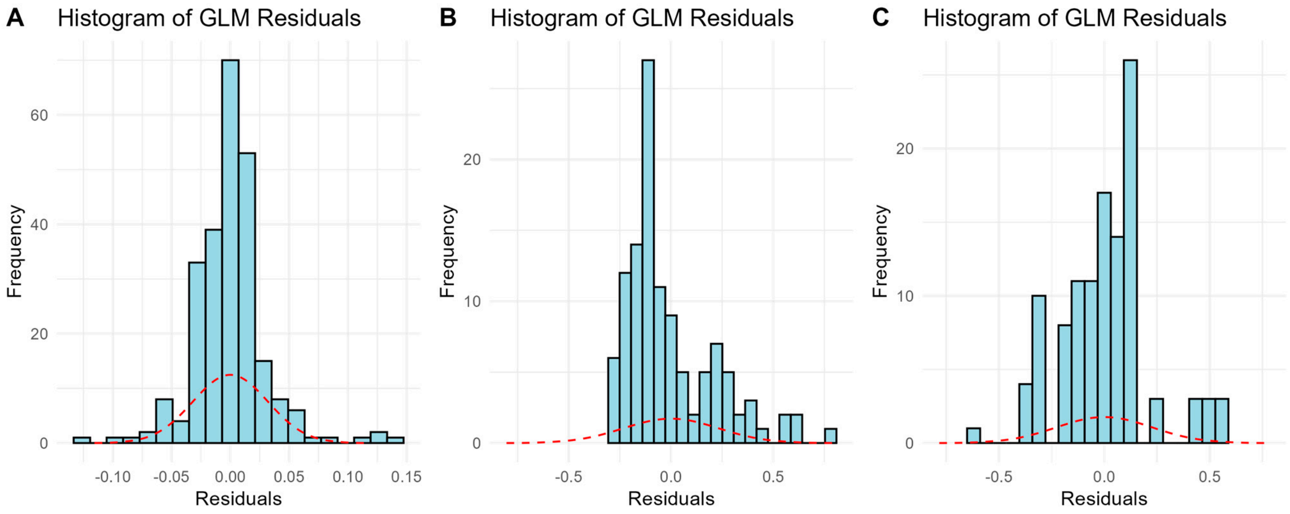

Figure S6. Residual distributions for (**A**) decomposition rate glm, (**B**) saprotroph diversity glm, and (**C**) pyrophilous fungal diversity glm.

Table S1. Pyrophilous fungi is known to occur in the United States, based on a comprehensive literature survey. Successional stage was divided into early (E), which means the fungi are initially found shortly after the fire (somewhere between hours and about 3 months) or later (L), meaning initially found between about 3 months and several years postfire. It was not possible to determine the exact successional timing of each fungus because it depends on environmental conditions such as snow and fire severity. Note that some early fungi persist for up to several years; early or later only refers to when they are initially found. Obligate pyrophilous fungi are those that require fire (Y) for reproduction. Few fungi have been studied for their fire-associated traits, but if we found information for these, it was recorded with a Y for Yes or an N for no and a ? if no information was found. Many pyrophilous fungi are also endophytic, with hosts ranging from Plants (P) to Bryophytes (B) to Lichens (L). The final column contains at least one reference that the fungus was found after fire.

Family	Pyrophilous Species	Synonyms	Successional Stage E = early, L = later	Obligate; Fire Required to Fruit	Fire Increases Fruiting	Fire-Adapted PyOM (Eat Charcoal)	Fire/Heat Tolerance Traits	Fruit Post-Fire to Escape	Ruderal (=Fast Growing or Sporulating, Weedy)	Bryophilous (B), Endophytic (P), Endolichenic (L)	Present After Fire References
Pyronemataceae	*Aleuria aurantia*		*l*	N [1]	Y [1,2]	?	?	?	Y [3]	not [4]	[1,3–5]
Pyronemataceae	*Anthracobia macrocystis*		*E*	Y [6]	Y [6]	Y [7]	?	?	?	B,L [4]	[6–10]
Pyronemataceae	*Anthracobia maurilabra*		*E*	Y [6]	Y [6,11]	Y [7]	?	?	?	B,L [4]	[6,8,11–13]
Pyronemataceae	*Anthracobia melaloma*		*E*	Y [6]	Y [6,11]	?	?	?	Y	B [14]	[3,5,6,8]
Pyronemataceae	*Anthracobia nitida*		*E*	Y [6]	Y [6]	?	?	?	?	B,L[4]	[5,8]
Pyronemataceae	*Anthracobia subatra*		*E*	Y [6]	Y [6]	?	?	?	?	?	[6,15]
Pyronemataceae	*Anthracobia tristis*		*E*	?	?	?	?	?	?	B,L [4]	[8,10,15,16]
Ascobolaceae	*Ascobolus carbonarius*		*E*	Y [6]	Y [6]	?	Y	?	?	P,L [4]	[3–6,9]
Pyronemataceae	*Ascobolus epimyces*		*l*	?	Y [4]	?	?	?	?	?	[4,10]
Pyronemataceae	*Ascobolus geophilus*		*l*	N [1]	N [1]	?	?	?	?	?	[1]
Pyronemataceae	*Ascobolus pusillus*		*l*	N [6]	Y [6]	?	?	?	?	?	[4]
Gelatinodiscaceae	*Ascocoryne cylichnium*		*l*	N [14]	?	?	?	?	Y	B [14]	[5,14,17]
Aspergillaceae	*Aspergillus australiensis*		*E*	?	?	N [7]	?	?	Y [18]	?	[7]
Aspergillaceae	*Aspergillus elsenburgensis*		*E*	?	?	N [7]	?	?	Y [18]	?	[7]

Aspergillaceae	*Aspergillus fumigatus*		*E*	?	?	N [7]	Y [7]	?	Y [7]	?	[7]
Aspergillaceae	*Aspergillus galapagensis*		*E*	?	?	Y [7]	Y [7]	?	Y [18]	?	[7]
Aspergillaceae	*Aspergillus papuensis*		*E*	?	?	Y [7]			Y [18]		[7]
Aspergillaceae	*Aspergillus udagawae*		*E*	?	?	Y [7]	?	?	Y [18]	?	[7]
Astraeaceae	*Astraeus pteridus*		*l*	?	Y [19]	?	Y [19]	?	?	?	[19]
Atheliaceae	*Athelia decipiens*		*l*	N [11]	Y [20]	?	?	?	?	?	[11,20]
Dothioraceae	*Aureobasidium pullulans*		*E*	?	Y [3,7]	N [7]	?	?	?	P [21]	[7]
Geminibasidiaceae	*Basidioascus undulatus*		*E*	?	Y [12]	?	Y	?	?	?	[9,12]
Pezizellaceae	*Bisporella citrina*	*Calycina citrina*	*l*	N [5]	Y [22]	?	?	?	?	?	[5]
Botryobasidiaceae	*Botryobasidium obtusisporum*		*E*	?	Y [20]	?	?	?	Y [23]	?	[18,20,23]
Sclerotiniaceae	*Botrytis cinerea*		*E*	N [24]	N [24]	?	?	?	Y [24]	P [25]	[10,24]
Phacidiaceae	*Bulgaria inquinans*		*L*	?	Y [5]	?	?	?	?	B [14]	[5]
Pyronemataceae	*Byssonectria fusispora*		*E*	?	Y [26]	?	?	?	Y [26]	?	[26,27]
Pyronemataceae	*Byssonectria seaveri*		*l*	?		?	?	?	?	?	[27]
Caolscyphaceae	*Caloscypha fulgens*		*l*	N [10]	N [10]	?	?	?	?	not [4]	[3,4,10]
Eurotiomycetidae	*Calyptrozyma*		*l*	N	Y [28,29]	?	?	?	?	?	[28,29]
Gloniaceae	*Cenococcum geophilum*		*l*	N [30]	N [30]	?	Y [31]	?	?	P [32,33]	[9]
Pyronemataceae	*Cheilymenia crucipila*		*l*	N [1]	N [1]	?	?	?	?		[1]
Pyronemataceae	*Cheilymenia vitellina*		*l*	?	?	?	?	?	?	not [4]	[4,5]
Moniliaceae	*Chrysosporium lobatum*		*l*	?		N [7]	?	?	?	?	[7]
Coltriciaceae	*Coltricia* aff. *perennis*		*l*	?	Y [11]	?	?	?	Y [11]	?	[11]
Coniochaetaceae	*Coniochaeta cipronana*		*E*	?	?	N [7]	?	?	?	P [34]	[7]
Coniochaetaceae	*Coniochaeta discospora*		*E*	Y [35]	Y [35]	?	Y [36]	?	?	?	[35,36]
Coniochaetaceae	*Coniochaeta hoffmannii*		*E*	?		N [7]	?	?	?	P [37]	[7]
Coniochaetaceae	*Coniochaeta lignaria*		*E*	?		?	?	?	?	B [14]	[14]
Coniochaetaceae	*Coniochaeta tetraspora*		*E*	Y [35]	Y [35]	?	Y [35]	?	?	P [38]	[35]
Psathyrellaceae	*Coprinellus angulatus*	*Tulosesus angulatus*	*E*	Y [6,11]	Y [6,11]	Y [18,39]	?	Y [18]	?	?	[5,6,11,18]
Psathyrellaceae	*Coprinellus micaceous*		*l*	N [11]	Y [11]	?	?	?	?	?	[11]
Psathyrellaceae	*Coprinellus radians*		*l*	?		Y [7]	?	?	?	?	[7]
Psathyrellaceae	*Coprinopsis jonesii*		*l*	?	Y [40]	?	?	?	?	?	[40]
Psathyrellaceae	*Coprinus disseminatus*		*l*	?	Y [5]	?	?	?	?	?	[5]
Psathyrellaceae	*Coprinus plicatilis*		*l*	?	Y [5]	?	?	?	?	?	[5]
Rickenellaceae	*Cotylidia undulata*		*l*	N [14]	Y [14]	?	?	?	?	B [14]	[14]
Crassisporiaceae	*Crassisporium funariophilum*		*E*	Y [41]		Y [18,39]	?	?	?	?	[18,39]
Incertae sedis	*Curvibasidium*		*E*	?		Y [18]	?	?	?	?	[18]
Hypoxylceae	*Daldinia loculata*		*l*	?	Y [42]	Y [18]	?	?	?	P [42]	[18,42]
Hypoxylceae	*Daldinia vernicosa*		*l*	?	Y [43]	?	?	?	?	P [44]	[15,43]

Pezizaceae	*Daleomyces petersii*	*Peziza petersii*	*E*	Y [16]	Y [11]	?	?	?	?	not [4]	[3,4,11,15,16]
Pezizaceae	*Daleomyces phillipsii*		*l*	?	Y [15]	?	?	?	?	?	[15,45]
Discinellaceae	*Discinella*		*l*	?		?	?	?	?	?	[3]
Steccherinacee	*Faerberia carbonaria*		*l*	Y [46]	Y [46]	?	?	?	?	?	[15,46]
Geminibasidiaceae	*Geminibasidium*		*E*	N [12]	Y [12]	?	Y [12]	?	?	P [47]	[9,12,28]
Geoglossaceae	*Geoglossum*		*l*	?	Y [28]	?	?	?	?	P [33]	[28]
Pyronemataceae	*Geopora arenicola*		*l*	?		?	?	?	?	?	[45]
Pyronemataceae	*Geopora cooperi*		*l*	?		?	?	?	?	P [48]	[48]
Pyronemataceae	*Geopyxis carbonaria*		*l*	Y [41]	Y [6]	Y [7]	?	?	?	P,L,B [4,14,49]	[4–7,9]
Pyronemataceae	*Geopyxis deceptiva*		*E*	N [49]	Y [49]	?	?	?	?	P [49]	[49]
Pyronemataceae	*Geopyxis delectans*		*E*	Y [49]	Y [49]	?	?	?	?	P [49]	[4]
Pyronemataceae	*Geopyxis majalis*		*?E*	N [49]		?	?	?	?	P,L [49]	[45]
Pyronemataceae	*Geopyxis rehmi*	*Geopyxis alpina in part*	*l*	N [49]		?	?	?	?	P,B [37]	[4,50]
Pezizaceae	*Geoscypha tenacella*	*Peziza tenacella*	*l*	?	Y [10]	?	?	?	?	P,L [4]	[4,10]
Pezizaceae	*Geoscypha violacea*	*Peziza praetervisa, Peziza violacea*	*E*	Y [6]	Y [6]	?	Y	?	?	P,L [4]	[3–6]
Hymenogastraceae	*Gymnopilus decipiens*		*l*	Y [14]	Y [11]	?	?	Y [18]	?	not	[11,14,18]
Discinaceae	*Gyromitra infula*		*l*	?	?	?	?	?	?	P,L [4]	[4,51]
Hymenogastraceae	*Hebeloma anthracophilum*		*l*	Y [9]	Y [9]	?	?	?	?	?	[5,9]
Hymenogastraceae	*Hebeloma nanum*		*l*	N [11]	Y [11]	?	?	?	?	?	[11]
Helvellacae	*Helvella crispa*		*l*	N [1]	N [1]	?	?	?	?	?	[1,5]
Helvellacae	*Helvella lacunosa*		*l*	N [1]	N [1]	?	?	?	?	?	[1,5]
Pleurotaceae	*Hohenbuehelia petaloides*		*l*	N [11]	Y [11]	?	?	?	?	?	[11]
Holtermaniales	*Holtermaniella festucosa*		*l*	?		N [7]	Y	?	?	P [52]	[7,53]
Hyaloscyphaceae	*Hyaloscypha (some)*		*l*	?	Y [54]	?	?	?	?	P [55]	[28]
Hygrophoraceae	*Hygrocybe conica*		*l*	N	Y [11]	?	?	?	?	P [56]	[11,54]
Hygrophoraceae	*Hygrocybe spadicea*		*l*	N	Y [11]	?	?	?	?	P [57]	[11]
Helotiaceae	*Hymenoscyphus serotinus*		*l*	N		?	?	?	?	L [58]	[5]
Inocybaceae	*Inocybe lacera*		*l*	N	Y [26]	?	?	?	?	?	[5]
Neoschizotheciaceae	*Jugulospora carbonaria*	*Strattonia carbonaria*	*l*	?	Y [16,35]	?	?	?	?	?	[15,35]
Hydnangiacee	*Laccaria laccata*		*l*	N [11]	Y [14]	?	?	?	?	B [14]	[5,11]
Hydnangiacee	*Laccaria trichodermophora*		*E*	N [11]	Y [11]	?	?	?	?	?	[11]
Lachnaceae	*Lachnum pygmaeum*		*l*	N	Y [59]	?	?	?	?	P [60]	[45]
Pyronemataceae	*Lamprospora carbonicola*		*l*	N	Y [61]	?	?	?	?	?	[3,45]

Pyronemataceae	*Lamprospora dictydiola*		*l*	N [6]	Y [6]	?	?	?	?	not [4]	[4,10]
Lyophyllaceae	*Lyophyllum anthracophilum*	*Tephrocybe anthracophila, Tephrocybe carbonaria,*	*l*	Y [6,11]	Y [6,11]	?	?	Y [18]	?	B [14]	[5,6,11,14,18,62]
Lyophyllaceae	*Lyophyllum atratum*	*Tephrocybe atrata*	*l*	Y [41]	Y [41]	Y [39]	?	?	?	?	[5,9,12,41]
Pezizaceae	*Marcelleina persoonii*	*Pulparia persoonii*	*l*	N [63]		?	?	?	?	?	Pfister pers. comm.
Morchellaceae	*Morchella capitata*	*Mel-9, Morchella exuberans*	*l*	Y [64]	Y [64]	?	?	?	?	?	[64]
Morchellaceae	*Morchella eximia*	*septimelata, Mel-7*	*l*	Y [64]	Y [64]	Y [7]	?	?	?	L [4]	[4,7,64,65]
Morchellaceae	*Morchella sextelata*	*Mel-6*	*l*	Y [14]	Y [64]		?	?		P [66]	[14,64]
Morchellaceae	*Morchella snyderi*	*Mel-12*	*l*	Y [41]	Y [41]	Y [41]	?	?	?	P [66]	[41]
Morchellaceae	*Morchella tomentosa*		*l*	Y [64]	Y [64]	?	?	?	?	?	[64]
Hydnaceae	*Multiclavula mucida*	*Clavaria mucida*	*l*	N [1]	Y [1]	?	?	?	?	?	[1]
Mycenaceae	*Mycena galericulata*		*E*	N [11]	Y [11]	?	?	Y [11]	?	?	[11]
Mycenaceae	*Mycena galopus*		*l*	N [67]	Y [5]	?	?	?	?	P [68]	[5]
Tricholomataceae	*Myxomphalia maura*	*Fayodia maura*	*E*	Y [69]	Y [1,3]	?	?	?	?	?	[1–3]
Pyronemataceae	*Neottiella hetieri*		*l*	N [1]	Y [1]	?	?	?	?	not [4]	[1,4]
Sordariaceae	*Neurospora calospora*	*Gelasinospora calospora*	*l*	Y [35]	Y [35]						[35]
Sordariaceae	*Neurospora crassa*		*l*	N [14]	Y [14]	Y [7]	?	Y [18]	Y [70]	B [14]	[2,3,14]
Sordariaceae	*Neurospora discreta*		*l*	Y [70]	Y [70]	Y [7]	Y	?	Y [70]	?	[7,70]
Sordariaceae	*Neurospora retispora*	*Gelasinospora reticulospora*	*E*	Y [16]	Y [35]	?	?	?	?	?	[3,16,35]
Sordariaceae	*Neurospora sitophila*		*l*	Y [70]	Y [70]				Y [70]		[70]
Gelatoporiaceae	*Obba rivulosa*	*Physisporinus rivulosus*	*l*	?	Y [20]		Y [71]				[20]
Pyronemataceae	*Octospora axilaris*		*E*	Y [16]	Y [16]	?	?	?	?	?	[16]
Pyronemataceae	*Octospora excipulata*		*l*	?	Y [15]	?	?	?	?	?	[15]
Pyronemataceae	*Octospora leucoloma*		*l*	N [1]	Y [1]	?	?	?	?	not [4]	[1,45]
Pyronemataceae	*Octospora rubens*		*l*	N [63]		?	?	?	?	?	[45]
Pyronemataceae	*Octospora rustica*		*l*	?		?	?	?	?	?	[45]
Omphalinaceae	*Omphalina pyxidata*		*l*	N [1]	Y [1]	?	?	?	?	?	[5]
Aspergillaceae	*Penicillium adametzii*		*E*	?	Y [7]	?	?	?	Y [18]	?	[7]
Aspergillaceae	*Penicillium chalabudae*		*E*	?	Y [7]	Y [7]	?	?	Y [18]	?	[7]
Aspergillaceae	*Penicillium chrysogenum*		*E*	?	Y [7]	Y [7]	Y [7]	?	Y [18]	P [72]	[7]
Aspergillaceae	*Penicillium citreosulfuratum*		*E*	?	Y [7]	?	?	?	Y [18]	?	[7]
Aspergillaceae	*Penicillium diabolicalicense*		*E*	?	Y [7]	?	?	?	Y [18]	?	[7]
Aspergillaceae	*Penicillium fagi*		*E*	?	Y [7]	?	?	?	Y [18]	?	[7]
Aspergillaceae	*Penicillium fellutanum*		*E*	?	Y [7]	N [7]	?	?	Y [18]	?	[7]
Aspergillaceae	*Penicillium glabrum*		*E*	?	Y [7]	Y [7]	?	?	Y [18]	P [37]	[7]

Aspergillaceae	*Penicillium meridianum*		*E*	?	Y [7]	?	?	?	Y [18]	?	[7]
Aspergillaceae	*Penicillium murcianum*		*E*	?	Y [7]	Y [7]	?	?	Y [18]	?	[7]
Aspergillaceae	*Penicillium oxalicum*		*E*	?	Y [7]	Y [7]	?	?	Y [18]	P [72]	[7]
Aspergillaceae	*Penicillium phillipense*		*E*	?	Y [7]	N [7]	?	?	Y [18]	?	[7]
Aspergillaceae	*Penicillium radiatolobatum*		*E*	?	Y [7]	?	?	?	Y [18]	?	[7]
Aspergillaceae	*Penicillium restrictum*		*E*	?	Y [7]	Y [7]	?	?	Y [18]	P [72]	[7]
Aspergillaceae	*Penicillium thomii*		*E*	?	Y [7]	N [7]	?	?	Y [18]	?	[7]
Pyronemataceae	*Perilachnea hemisphaerioides*	*Humaria hemisphaerioides*	*l*	Y [6]	Y [6]	?	?	?	?	P,L [4]	[3–6,45]
Pezizaceae	*Peziza acanthodictya*		*l*	?		?	?	?	?	P,L [4]	[4]
Pezizaceae	*Peziza arvernensis*		*l*	N [73]	?	?	?	?	?	?	[45,73]
Pezizaceae	*Peziza atrovinosa*		*l*	N [6]	Y [6]	?	?	?	?	not [4]	[4,5]
Pezizaceae	*Peziza echinospora*	*Peziza anthracophila*	*l*	Y [14]	Y [6,11]	Y [41]	?	?	?	P [4],B [14]	[5–7,10,11]
Pezizaceae	*Peziza lobulata*		*l*	Y [11]	Y [11]	?	?	?	?	not [4]	[4,5,11]
Pezizaceae	*Peziza michelii*		*l*	N [11]	Y [11]	?	?	?	?		[11]
Pezizaceae	*Peziza ostracoderma*		*l*	N [74]		?	?	?	?	P [4]	[4]
Pezizaceae	*Peziza pseudoviolacea*		*E*	Y [16]	Y [16]	?	?	?	?		[16]
Pezizaceae	*Peziza saccardoana*		*l*	?	?	?	?	?	?	not [14]	[14,45]
Pezizaceae	*Peziza sepiatra*		*l*	?	Y [75]	?	?	?	?	?	[45,75]
Pezizaceae	*Peziza subvesiculosa*		*l*	?		?	?	?	?	?	[45]
Pezizaceae	*Peziza varia*		*l*	N [11]		?	?	?	?	P,L [4]	[11,45]
Strophariaceae	*Pholiota brunnescens*	*Pholiota luteobadia*	*E*	Y [76]	Y [76]	Y [7]	?	?	?	?	[7,76]
Strophariaceae	*Pholiota castanea*		*l*	Y [14]	Y [14]	?	?	?	?	B [14]	[14,76]
Strophariaceae	*Pholiota highlandensis*	*Pholiota carbonaria, Pholiota carbonicola*	*E*	Y [6,11]	Y [6,11]	Y	?	Y [18]	?	B [14]	[5,6,9,14,18,76]
Strophariaceae	*Pholiota molesta*	*Pholiota subsaponacea*	*l*	Y [41,76]		Y [39]	?	?	?	?	[39,76]
Pezizaceae	*Phylloscypha phyllogena*	*Peziza phyllogena*	*l*	*?*	Y [11]	?	?	?	?	?	[11]
Pezizaceae	*Plicaria acanthodictya*		*l*	?	?	?	?	?	?	P,L,B [4,14]	[4,45]
Pezizaceae	*Plicaria carbonaria*	*Peziza anthracina*	*l*	Y [6,11]	Y [6]	?	?	?	?	B [14]	[6,11]
Pezizaceae	*Plicaria endocarpoides*	*Peziza endocarpoides, Plicaria leiocarpa*	*l*	N [6]	Y [6]	?	?	?	?	P,L [4]	[3,4,6,11]
Pezizaceae	*Plicaria lundelii*		*l*	?		?	?	?	?	L [4]	[5]
Pezizaceae	*Plicaria trachycarpa*	*Peziza trachycarpa*	*l*	Y [6]	Y [6]	?	?	?	?	?	[6,11]
Psathyrellaceae	*Psathyrella pennata*	*Psathyrella carbonicola*	*E*	Y [11]	Y [11]	?	?	?	?	B [14]	[5,11,14]

Discinaceae	*Pseudoverpa anthracobia*	*Gyromitra anthracobia*	*L*	Y [77]	[77]	?	?	?	?	?	[45,77]
Pyronemataceae	*Pulvinula archeri*		*E*	Y [16]	Y [16]	?	?	?	?	P,L [4]	[3,4,10]
Pyronemataceae	*Pulvinula carbonaria*	*Lamprosora carbonaria*	*E*	Y [1]		?	?	?	?	?	[1]
Pyronemataceae	*Pulvinula laeterubra*		*l*	?		?	?	?	?	?	[45]
Pyronemataceae	*Pulvinula neotropica*		*l*	[78]	[78]	?	?	?	?	?	[78]
Pyronemataceae	*Pulvinula tetraspora*		*l*	?		?	?	?	?	?	[4]
Pezizaceae	*Purpureodiscus subisabellinus*		*l*	?		?	?	?	?	?	[45]
Pyronemataceae	*Pyronema*		*E*	?		?	?	?	?	?	[9,28]
Pyronemataceae	*Pyronema domesticum*		*E*	Y [14,41]	Y [14]	Y [79]	Y	?	?	B [14]	[5,7,9,14,41,79]
Pyronemataceae	*Pyronema omphalodes*	*Pyronema confluens*	*E*	Y [6,41]	Y [6,11]	Y [41]	?	?	?	P,L [4]	[4–7,9,41]
Pyropyxidaceae	*Pyropyxis rubra*		*l*	?		?	?	?	?	P,L,B [4,14]	[4,10,80]
Aspergillaceae	*Rasamsonia columbensis*		*l*	?		N [7]	Y [7]	?	?	?	[7]
Rhizinaceae	*Rhizina undulata*		*l*	Y [11,14]	Y [11,14]	?	?	?	?	P,L,B [4,14]	[3,5,11,15]
Rhizopogonaceae	*Rhizopogon olivaceotinctus*	*Alpova olivaceotinctus*	*l*	Y [81]		?	Y [81]	?	?	?	[9,18,23,81]
Rhizopogonaceae	*Rhizopogon roseolus*		*l*	N [82]	Y [82]	?	Y [82]	?	Y [82]	?	[9]
Sporidiobolaceae	*Rhodosporidiobolus*		*l*	Y [28]	Y [28]		?		?	P [83]	[28]
Pyronemataceae	*Rhodotarzetta rosea*	*Tarzetta rosea*	*l*	N [84]		?	?	?	?	not [4]	[4,10]
Schizophyllaceae	*Schizophylum commune*		*l*	N	Y [85]	?	?	?	Y [85]	P [86]	[3,5]
Pyronemataceae	*Scutellinia*		*l*	N [84]	Y [48]	?	?	?	?	P,L [4]	[9,12]
Pyronemataceae	*Scutellinia scutellata*		*l*	N [84]		?	?	?	Y [84]	P,L [4]	[3–5]
Piskurozymaceae	*Solicoccozyma*		*E*	?	Y [28,87]	?	?	?	?	P [88]	[28,87]
Pyronemataceae	*Sphaerosporella "brunnea"*	*Trichophaea brunnea*	*l*	N [13]	Y [6,13]	?	?	?		P,L [4]	[4,6,10,13]
Pyronemataceae	*Sphaerosporella hinnulea*		*l*	?	?	?	?	?	?	P,L [4]	[4,9,13]
Thelephoraceae	*Thelephora palmata*		*l*	N [11]	?	?	?	?	?	?	[5,11]
Thelephoraceae	*Thelephora terrestris*		*l*	N [11]	?					B [14]	[11,14]
Thelephoraceae	*Thelephora vialis*		*l*	N [11]	?						[11]
Pyronemataceae	*Tricharina gilva*		*l*	N [89]	Y [89]	?	?	?	?	P,L [4]	[4,12,89]
Pyronemataceae	*Tricharina praecox*	*Ascorhizoctonia praecox*	*E*	Y [14]	Y [14]	Y [41]	Y	?	?	P,L,B [4,14]	[4,7,10,41,45,90]
Hypocreaceae	*Trichoderma viride*		*l*	?	Y [18]	?	?	?	Y [18]	P [91]	[18]
Pyronemataceae	*Trichophaea abundans*	*Anthracobia humillima*	*l*	Y	Y [43]	?	Y	?	?	P,L [4]	[4,9,10,43]
Pyronemataceae	*Trichophaea gregaria*		*l*	N [1]	Y [1]	?	?	?	?	?	[1,4]

Pyronemataceae	*Trichophaea woolhopeia*		*l*	N [92]		?	?	?	?	P [84]	[45]
Pezizaceae	*Velenovskya vacini*		*l*	N	Y [89]	?	?	?	?	?	[45,89]
Otidiaceae	*Warcupia terrestris*		*l*	N [93]	Y [69]		?	?	?	?	[69]
Pyronemataceae	*Wilcoxina gregaria*	*Humaria gregaria*	*l*	N [6]	Y [6]		?	?	?	?	[6]
Pyronemataceae	*Wilcoxina mikolae*		*E*	Y [41]	Y [41]	Y [41]	Y [81]	?	?	?	[41]
Pyronemataceae	*Wilcoxina rehmii*		*l*	N	Y [94]	?	?	?	?	P [48,90]	[9,94]
Boletaceae	*Xerocomellus*		*l*	N	Y [28]	?	?	?	?		[28]
Boletaceae	*Xerocomellus atropurpureus*		*l*	N	Y [19]	?	?	?	?	?	[19]

Abbreviations: E = early, l = later, Y = yes, N = no, P = Plant, B = Bryophyte, L = Lichen, ? = no information found.

Table S2. C:N response to decomposition rate, burn and autoclave treatments.

Nutrient Response				
	**Estimate**	**Std Error**	**t Value**	**Pr (>F)**
Decomp rate	−106.132	40.901	−2.595	0.01
Burn	−7.361	5.365	−1.372	0.18
Autoclaved	1.004	5.11	0.197	0.85
Fresh.Weight	11.244	5.947	1.891	0.07
Site	−9.337	3.556	−2.626	0.01
Burn: Autoclaved	−3.032	6.861	−0.442	0.66

fresh litter weight, and site. C:N declined with decomposition rate.

Table S3. Permanova response for saprotroph and pyrophile communities. Differences in fungal community composition between burn and autoclave treatments (both separately and interacting), along with site. Saprotroph community composition was not significantly different between sites or treatments; however, pyrophilous fungal communities differed between autoclave treatments and sites.

Permanova										
	**Saprotrophs**					**Pyrophiles**				
	Df	SumofSqs	R2	F	Pr (>F)	Df	SumofSqs	R2	F	Pr (>F)
Burn	1.000	1.238	0.010	0.958	0.582	1.000	1.264	0.024	1.481	0.168
Autoclaved	1.000	1.332	0.010	1.031	0.364	1.000	2.274	0.042	2.666	0.010
Site	2.000	2.296	0.018	0.889	0.894	2.000	4.789	0.089	2.807	0.001
Burn: Autoclaved	1.000	1.306	0.010	1.011	0.417	1.000	1.039	0.019	1.218	0.276
Residual	94.000	121.420	0.952			52.000	44.360	0.826		
Total	99.000	127.592	1.000			57.000	53.726	1.000		

Decomposition Rates—No Pisgah.				
	**Estimate**	**Std Error**	**t Value**	**Pr (>F)**
Burn	−0.019	0.008	−2.516	0.013

Autoclaved	−0.018	0.007	−2.521	0.013
Months.out	−0.003	0.001	−4.339	2.59E−05
Fresh.Weight	0.008	0.009	0.882	0.38
Site-Spire	−0.010	0.006	−1.878	0.06
Burn: Autoclaved	0.006	0.011	0.565	0.57

Table S4. Decomposition rate generalized linear model, with the Pisgah sites removed.

Literature Cited

1. Lisiewska, M., *Macrofungi on special substrates*, in *Fungi in vegetation science*. 1992, Springer. p. 151–182.
2. Dix, N.J. and J. Webster, *Phoenicoid fungi*, in *Fungal Ecology*. 1995, Springer Netherlands: Dordrecht. p. 302–321.
3. Carpenter, S.E., J.M. Trappe, and J. Ammirati, *Observations of fungal succession in the Mount St. Helens devastation zone, 1980-1983.* Canadian Journal of Botany-Revue Canadienne De Botanique, 1987. 65(4): p. 716–728.
4. Healy, R., et al., *Endophytism and endolichenism in Pezizomycetes: the exception or the rule?* New Phytologist, 2022. 233(5): p. 1974–1983.
5. Adamczyk, J.J., et al., *Factors shaping communities of pyrophilous macrofungi in microhabitats destroyed by illegal campfires.* Fungal Biology, 2012. 116(9): p. 995–1002.
6. Petersen, P.M., *Fireplace fungi in an arctic area: middle west Greenland.* Friesia, 1974. 10(4-5): p. 270–280.
7. Enright, D.J., *Unraveling fire adaptation traits in pyrophilous bacteria and fungi*, in *Microbiology*. 2024, University of California Riverside: Riverside, California.
8. Larsen JR, H.J., *The genus Anthracobia Boudier (Pezizales, Ascomycetes)*, in *Botany*. 1976, Oregon State University: Corvallis, Oregon.
9. Fox, S., et al., *Fire as a driver of fungal diversity — A synthesis of current knowledge.* Mycologia, 2022. 114(2): p. 215–241.
10. Egger, K. and J. Paden, *Pathogenicity of postfire ascomycetes (Pezizales) on seeds and germinants of lodgepole pine.* Canadian Journal of Botany, 1986. 64: p. 2368–2371.
11. Hughes, K.W., et al., *Pyrophilous fungi detected after wildfires in the Great Smoky Mountains National Park expand known species ranges arid biodiversity estimates.* Mycologia, 2020. 112(4): p. 677–698.
12. Enright, D., et al., *Mega-fire in redwood tanoak forest reduces bacterial and fungal richness and selects for pyrophilous taxa that are phylogenetically conserved.* Molecular Ecology, 2022. 31(8): p. 2475–2493.
13. Hughes, K.W., et al., *Secret lifestyles of pyrophilous fungi in the genus Sphaerosporella.* American Journal of Botany, 2020. 107(6): p. 876–885.
14. Raudabaugh, D.B., et al., *Where are they hiding? Testing the body snatchers hypothesis in pyrophilous fungi.* Fungal Ecology, 2020. 43: p. https://doi.org/10.1016/j.funeco.2019.100870.
15. Van Vooren, N., et al., *La fonge carbonicole. Résultats du suivi de sites incendiés en France en 2022.* Ascomycete.org, 2022. 66.
16. Claridge, A.W., J.M. Trappe, and K. Hansen, *Do fungi have a role as soil stabilizers and remediators after forest fire?* Forest Ecology and Management, 2009. 257(3): p. 1063–1069.
17. Vasiliauskas, R. and J. Stenlid, *Fungi inhabiting stems of Picea abies in a managed stand in Lithuania.* Forest ecology and management, 1998. 109(1-3): p. 119–126.
18. Hopkins, J.R. and A.E. Bennett, *Leveraging traits for insight into the fungal ecology of burned ecosystems.* Ecosphere, 2024. 15(11): p. e70008.
19. Roy, B., et al., *Macrofungi at H. J. Andrews.* in prep.
20. Penttilä, R. and H. Kotiranta, *Short-term effects of prescribed burning on wood-rotting fungi.* Silva Fennica, 1996. 30(4): p. 399–419.
21. Parsa, S., et al., *Fungal endophytes in germinated seeds of the common bean, Phaseolus vulgaris.* Fungal biology, 2016. 120(5): p. 783–790.
22. Kebli, H., et al., *Impact of harvesting intensity on wood-inhabiting fungi in boreal aspen forests of Eastern Canada.* Forest Ecology and Management, 2012. 279: p. 45–54.
23. Glassman, S.I., et al., *Ectomycorrhizal fungal spore bank recovery after a severe forest fire: some like it hot.* Isme Journal, 2016. 10(5): p. 1228–1239.
24. Rojas, D.S. and G.S. Gilbert, *The response of Botrytis cinerea to fire in a coast redwood forest.* International Journal of Plant Biology, 2024. 15(1): p. 94–101.
25. Van Kan, J.A., M.W. Shaw, and R.T. Grant-Downton, *Botrytis species: relentless necrotrophic thugs or endophytes gone rogue?* Molecular Plant Pathology, 2014. 15(9): p. 957–961.
26. Orumaa, A., et al., *Long-term effects of forest fires on fungal community and soil properties along a hemiboreal Scots pine forest fire chronosequence.* Science of The Total Environment, 2022. 851: p. 158173.
27. Pfister, D.H., *A synopsis of the North American species of Byssonectria (Pezizales) with comments on the ontogeny of two species.* Mycologia, 1993. 85(6): p. 952–962.
28. Fischer, M.S., et al., *Prescribed fire selects for a pyrophilous soil sub-community in a northern California mixed conifer forest.* Environmental Microbiology, 2023. 25(11): p. 2498–2515.
29. Whitman, T., et al., *Soil bacterial and fungal response to wildfires in the Canadian boreal forest across a burn severity gradient.* Soil Biology and Biochemistry, 2019. 138: p. 107571.
30. Hughes, K.W., et al., *Post-fire Quercus mycorrhizal associations are dominated by Russulaceae, Thelephoraceae, and Laccaria in the southern Appalachian Mountains.* Mycological Progress, 2025. 24(1): p. 1–18.
31. Li, M., et al., *The transcriptional responses of ectomycorrhizal fungus, Cenococcum geophilum, to drought stress.* Journal of Fungi, 2022. 9(1): p. 15.
32. LoBuglio, K., *Cenococcum*, in *Ectomycorrhizal fungi key genera in profile*. 1999, Springer. p. 287–309.
33. Bergemann, S. and M. Garbelotto, *High diversity of fungi recovered from the roots of mature tanoak (Lithocarpus densiflorus) in northern California.* Botany, 2006. 84(9): p. 1380–1394.
34. Coronado-Ruiz, C., et al., *Two new cellulolytic fungal species isolated from a 19th-century art collection.* Scientific Reports, 2018. 8(1): p. 7492.

35. Wicklow, D.T., *Fire as an environmental cue initiating ascomycete development in a tallgrass prairie.* Mycologia, 1975. 67(4): p. 852–862.
36. Wicklow, D.T., *Microfungal populations in surface soils of manipulated prairie stands.* Ecology, 1973. 54(6): p. 1302–1310.
37. Kiser, J., H. Daniels, and J. Scrivani, *Isolation of endophytic fungi from Douglas-Fir (Pseudotsuga menziesii) foliage with bioprospecting potential for natural pharmaceuticals.* Current Trends in Forest Research, 2019. 3(01): p. 2638–0013.100035.
38. Petrini, O. and P. Fisher, *A comparative study of fungal endophytes in xylem and whole stem of Pinus sylvestris and Fagus sylvatica.* Transactions of the British Mycological Society, 1988. 91(2): p. 233–238.
39. Steindorff, A.S., et al., *Comparative genomics of pyrophilous fungi reveals a link between fire events and developmental genes.* Environmental microbiology, 2021. 23(1): p. 99–109.
40. Noordeloos, M.E. and U. CB, *Type studies in Coprinus subsect. Alachuani.* Proceedings of the Koninklijke Nederlandse Akademie Van Wetenschappen-Biological Chemical Geological Physical and Medical Sciences, 1996. 99: p. 105–124.
41. Steindorff, A.S., et al., *Diversity of genomic adaptations to the post-fire environment in Pezizales fungi points to crosstalk between charcoal tolerance and sexual development.* New Phytologist, 2022. 236(3): p. 1154–1167.
42. Johannesson, H., M. Gustafsson, and J. Stenlid, *Local population structure of the wood decay ascomycete Daldinia loculata.* Mycologia, 2001. 93(3): p. 440–446.
43. Gierczyk, B., et al., *Contribution to the knowledge of fungi of the Kampinos National Park (Poland) with particular emphasis on the species occurring in burnt places.* Acta Mycologica, 2017. 52(1).
44. Yu, M., et al., *Structures and Biological Activities of Secondary Metabolites from Daldinia spp.* Journal of Fungi, 2024. 10(12): p. 833.
45. Dougoud, R., *Clé des Discomycètes carbonicoles.* Documents mycologiques, 2001. 30(120): p. 15–29.
46. Gulde, G., *Three new agarics in Norway.* Agarica, 2011. 30: p. 103–109.
47. Diez-Hermano, S., et al., *Soil mycobiome and forest endophytic fungi: Is there a relationship between them?* Forest Ecology and Management, 2024. 562: p. 121924.
48. Fujimura, K., et al., *Pezizalean mycorrhizas and sporocarps in ponderosa pine (Pinus ponderosa) after prescribed fires in eastern Oregon, USA.* Mycorrhiza, 2005. 15: p. 79–86.
49. Wang, X.H., S. Huhtinen, and K. Hansen, *Multilocus phylogenetic and coalescent-based methods reveal dilemma in generic limits, cryptic species, and a prevalent intercontinental disjunct distribution in Geopyxis (Pyronemataceae s. l., Pezizomycetes).* Mycologia, 2016. 108(6): p. 1189–1215.
50. Turnau, K., *Investigations on post-fire Discomycetes: Geopyxis rehmii sp. nov. and G. carbonaria (Alb. & Schw. ex Fr.) Sacc.* Nova Hedwigia, 1985. 40(1-4): p. 157–170.
51. Egger, K.N., *Substrate hydrolysis patterns of post-fire ascomycetes (Pezizales).* Mycologia, 1986. 78(5): p. 771–780.
52. Marchi, M., et al., *Unexplored Yeast diversity in Seed Microbiota.* bioRxiv, 2024: p. 2024.11. 27.625647.
53. Caiafa, M.V., et al., *Distinct fungal and bacterial responses to fire severity and soil depth across a ten-year wildfire chronosequence in beetle-killed lodgepole pine forests.* Forest Ecology and Management, 2023. 544: p. 121160.
54. Bruns, T.D., et al., *A simple pyrocosm for studying soil microbial response to fire reveals a rapid, massive response by Pyronema species.* PLOS ONE, 2020. 15(3): p. e0222691.
55. Vohník, M. and M. Réblová, *Fungi in hair roots of Vaccinium spp.(Ericaceae) growing on decomposing wood: colonization patterns, identity, and in vitro symbiotic potential.* Mycorrhiza, 2023. 33(1): p. 69–86.
56. Halbwachs, H., et al., *Hyphae of waxcap fungi colonise plant roots.* Fungal Ecology, 2013. 6(6): p. 487–492.
57. Voyron, S., et al., *Fine-scale spatial distribution of orchid mycorrhizal fungi in the soil of host-rich grasslands.* New Phytologist, 2017. 213(3): p. 1428–1439.
58. Pressel, S., et al., *A novel ascomycetous endophytic association in the rhizoids of the leafy liverwort family, Schistochilaceae (Jungermanniidae, Hepaticopsida).* American Journal of Botany, 2008. 95(5): p. 531–541.
59. Durán, M., et al., *Disruption of traditional grazing and fire regimes shape the fungal endophyte assemblages of the tall-grass Brachypodium rupestre.* Frontiers in Microbiology, 2021. 12: p. 679729.
60. Lou, H., et al., *Lingonberry (Vaccinium vitis-idaea L.) interact with Lachnum pygmaeum to mitigate drought and promote growth.* Frontiers in Plant Science, 2022. 13: p. 920338.
61. Vega, M., et al., *Lamprospora densireticulata sp. nov., L. dictydiola and L. carbonicola (Pyronemataceae, Pezizales)—three very similar species from very different hosts and habitats.* Mycological Progress, 2019. 18: p. 1013–1026.
62. Salo, K., T. Domisch, and J. Kouki, *Forest wildfire and 12 years of post-disturbance succession of saprotrophic macrofungi (Basidiomycota, Ascomycota).* Forest Ecology and Management, 2019. 451: p. 117454.
63. Schumacher, T., *Notes on taxonomy, ecology, and distribution of.* Botany, 1979. 1: p. 53–83.
64. Kuo, M., et al., *Taxonomic revision of true morels (Morchella) in Canada and the United States.* Mycologia, 2012. 104(5): p. 1159–1177.
65. Richard, F., et al., *True morels (Morchella, Pezizales) of Europe and North America: evolutionary relationships inferred from multilocus data and a unified taxonomy.* Mycologia, 2015. 107(2): p. 359–382.
66. Baynes, M., et al., *A novel plant–fungal mutualism associated with fire.* Fungal Biology, 2012. 116(1): p. 133–144.
67. Perry, B.A., *A taxonomic investigation of Mycena in California*, in *Ecology and Systematic Biology*. 2002, San Francisco State University: San Francisco State University.
68. Thoen, E., et al., *In vitro evidence of root colonization suggests ecological versatility in the genus Mycena.* New Phytologist, 2020. doi: 10.1111/nph.16545.

69. Philpott, T.J., et al., *Low-severity wildfire prevents catastrophic impacts on fungal communities and soil carbon stability in a fire-affected Douglas-fir ecosystem.* Geoderma, 2025. 454: p. 117189.
70. Jacobson, D.J., et al., *Neurospora in temperate forests of western North America.* Mycologia, 2004. 96(1): p. 66–74.
71. Hildén, K., et al., *Novel thermotolerant laccases produced by the white-rot fungus Physisporinus rivulosus.* Applied Microbiology and Biotechnology, 2007. 77: p. 301–309.
72. Toghueo, R.M.K. and F.F. Boyom, *Endophytic Penicillium species and their agricultural, biotechnological, and pharmaceutical applications.* 3 Biotech, 2020. 10(3): p. 107.
73. Lantieri, A., G. Medardi, and P. Alvarado, *Morphological and phylogenetic clarification of Peziza arvernensis, P. pseudovesiculosa, P. pseudosylvestris, and P. domiciliana.* Mycotaxon, 2016. 131(4): p. 827–839.
74. Hennebert, G. and R.P. Korf, *The peat mould, Chromelosporium ollare, conidial state of Peziza ostracoderma, and its misapplied names, Botrytis crystallina, Botrytis spectabilis, Ostracoderma epigaeum and Peziza atrovinosa.* Mycologia, 1975. 67(2): p. 214–240.
75. Nanagulyan, S. and L. Margaryan, *Mycobiota of Armenia*, in *Biodiversity of Armenia*. 2023, Springer. p. 143–164.
76. Matheny, P.B., et al., *Revision of pyrophilous taxa of Pholiota described from North America reveals four species—P. brunnescens, P. castanea, P. highlandensis, and P. molesta.* Mycologia, 2018. 110(6): p. 997–1016.
77. Crous, P., et al., *Fungal Planet description sheets: 716-784.* Persoonia, 2018. 40: p. 240–393.
78. Pfister, D.H., *A synopsis of the genus Pulvinula.* Occasional papers of the Farlow Herbarium of cryptogamic botany, 1976: p. 1–19.
79. Fischer, M.S., et al., *Pyrolyzed substrates induce aromatic compound metabolism in the post-fire fungus, Pyronema domesticum.* Frontiers in microbiology, 2021. 12: p. 729289.
80. Egger, K.N., *Pyropyxis, a new pyrophilous operculate discomycte with a Dichobotrys anamorph.* Canadian Journal of Botany-Revue Canadienne De Botanique, 1984. 62(4): p. 705–708.
81. Peay, K.G., M. Garbelotto, and T.D. Bruns, *Spore heat resistance plays an important role in disturbance-mediated assemblage shift of ectomycorrhizal fungi colonizing Pinus muricata seedlings.* Journal of Ecology, 2009. 97(3): p. 537–547.
82. Kipfer, T., et al., *Ectomycorrhiza succession patterns in Pinus sylvestris forests after stand-replacing fire in the Central Alps.* Oecologia, 2011. 167: p. 219–228.
83. Glushakova, A. and A. Kachalkin, *Endophytic yeasts in Malus domestica and Pyrus communis fruits under anthropogenic impact.* Microbiology, 2017. 86: p. 128–135.
84. Hansen, K., et al., *A phylogeny of the highly diverse cup-fungus family Pyronemataceae (Pezizomycetes, Ascomycota) clarifies relationships and evolution of selected life history traits.* Molecular Phylogenetics and Evolution, 2013. 67(2): p. 311–335.
85. Motiejūnaitė, J., et al., *Early fungal community succession following crown fire in Pinus mugo stands and surface fire in Pinus sylvestris stands.* European journal of forest research, 2014. 133: p. 745–756.
86. Kleijburg, F.E. and H.A. Wösten, *The versatility of Schizophyllum commune in nature and application.* Fungal Biology Reviews, 2025. 53: p. 100431.
87. Yang, T., et al., *Distinct fungal successional trajectories following wildfire between soil horizons in a cold-temperate forest.* New Phytologist, 2020. 227(2): p. 572–587.
88. Li, S., et al., *Endophytic fungal and bacterial microbiota shift in rice and barnyardgrass grown under co-culture condition.* Plants, 2022. 11(12): p. 1592.
89. Van Vooren, N., U. Lindemann, and R. Healy, *Emendation of the genus Tricharina (Pezizales) based on phylogenetic, morphological and ecological data.* Ascomycete. org, 2017. 9(4): p. 101–123.
90. Gervers, K.A., et al., *Crown closure affects endophytic leaf mycobiome compositional dynamics over time in Pseudotsuga menziesii var. menziesii.* Fungal Ecology, 2022. 57: p. 101155.
91. Poveda, J., *Trichoderma as biocontrol agent against pests: New uses for a mycoparasite.* Biological Control, 2021. 159.
92. Tedersoo, L., T.W. May, and M.E. Smith, *Ectomycorrhizal lifestyle in fungi: global diversity, distribution, and evolution of phylogenetic lineages.* Mycorrhiza, 2010. 20(4): p. 217–263.
93. Paden, J.W. and J.V. Cameron, *Morphology of Warcupia terrestris, a new ascomycete genus and species from soil.* Canadian Journal of Botany, 1972. 50(5): p. 999–1001.
94. Pulido-Chavez, M.F., et al., *High-severity wildfire reduces richness and alters composition of ectomycorrhizal fungi in low-severity adapted ponderosa pine forests.* Forest Ecology and Management, 2021. 485: p. 118923.
